# Supplementary material for: Aberrant causal inference and presence of a compensatory mechanism in autism spectrum disorder
Source: eLife. 2022 May 17;11:e71866. doi: 10.7554/eLife.71866 (PMC9170250; doi:10.7554/eLife.71866)
Supplement: Supplementary file 2. — The table indexes each ASD participant by a unit ID, and indicates in which experiment did each participant take part in. Green indicates that the participant took part in the given experiment, while red indicates that they did not. An orange box indicates that the participant took part in the experiment, but their data was excluded in presentation of the empirical results (but not the modeling of individual subjects, as indicated in the main text). Experiment 1 is the unisensory discrimination task and audio-visual cue combination that does not require causal inference (i.e. imperceptible disparities). Experiment 2 is the audio-visual implicit causal inference experiment. Experiment 3 is the explicit causal inference experiment with spatial disparities. Experiment 4 is the explicit causal inference experiment with temporal disparities. Experiment 5 is the visual heading discrimination task during concurrent object-motion. [file elife-71866-supp2.docx]

**Supplementary File 2. ASD participants.** The table indexes each ASD participant by a unit ID, and indicates in which experiment did each participant take part in. Green indicates that the participant took part in the given experiment, while red indicates that they did not. An orange box indicates that the participant took part in the experiment, but their data was excluded in presentation of the empirical results (but not the modeling of individual subjects, as indicated in the main text). Experiment 1 is the unisensory discrimination task and audio-visual cue combination that does not require causal inference (i.e., imperceptible disparities). Experiment 2 is the audio-visual implicit causal inference experiment. Experiment 3 is the explicit causal inference experiment with spatial disparities. Experiment 4 is the explicit causal inference experiment with temporal disparities. Experiment 5 is the visual heading discrimination task during concurrent object-motion.
