# Supplementary material for: Aberrant causal inference and presence of a compensatory mechanism in autism spectrum disorder
Source: eLife. 2022 May 17;11:e71866. doi: 10.7554/eLife.71866 (PMC9170250; doi:10.7554/eLife.71866)
Supplement: Supplementary file 3. [file elife-71866-supp3.docx]

**Supplementary File 3. Priors distributions over model parameters**

| **Parameter** | **Prior distribution** | **Notes** |
| --- | --- | --- |
| Unisensory Lapse rate ($p_{\text{lapse rate}}^{\text{uni}}$) | $p_{\text{lapse rate}}^{\text{uni}}\sim Beta(1,5)$ | Weakly informative prior preferring lower lapse rates |
| Unisensory Lapse bias ($p_{\text{lapse bias}}^{\text{uni}}$) | $p_{\text{lapse bias}}^{\text{uni}}\sim Beta(1.25,1.25)$ | Weakly informative prior preferring unbiased lapses |
| Lapse rate in implicit and explicit causal inference tasks ($p_{\text{lapse rate}}^{\text{multi}}$) | $p_{\text{lapse rate}}^{\text{multi}}\sim Sigmoid[\ln\frac{p_{\text{lapse rate}}^{\text{uni}}}{1-p_{\text{lapse rate}}^{\text{uni}}}+\zeta$]  where  $\zeta\sim\mathcal{N(}{0,0.5}^{2})$  and  $Sigmoid\left( x \right)=\frac{1}{1+exp(-x)}$ | Hierarchical: This prior allows the model to hierarchically infer a lapse rate that is consistent across experiments unless the data suggests otherwise |
| Lapse bias in implicit & explicit causal inference tasks ($p_{\text{lapse bias}}^{\text{multi}}$) | $p_{\text{lapse bias}}^{\text{multi}}\sim Sigmoid[\ln\frac{p_{\text{lapse bias}}^{\text{multi}}}{1-p_{\text{lapse bias}}^{\text{multi}}}+\zeta$]  where  $\zeta\sim\mathcal{N(}{0,0.5}^{2})$ | Hierarchical (see above) |
| Auditory and visual location prior mean ($\mu_{a},\mu_{v}$) | $\mu_{a}\sim\mathcal{N(}{0,10}^{2})$  $\mu_{v}\sim\mathcal{N(}{0,10}^{2})$ | Weakly informative prior preferring no perceptual bias |
| Auditory location sensory noise standard deviation ($\sigma_{a}$) | $\ln\sigma_{a}\sim\mathcal{N(}{2.21,0.69}^{2})$ | Chosen to explore the full range of psychometric curves. The parameters were chosen by finding values of $\sigma_{a}$ for limiting psychometric curves (shallow and steep) for the range of values used in experiments. The limiting values were translated to parameters of the lognormal distribution such that 90% of the mass lied within the range. We verified that the results were insensitive to the exact value of the prior by varying the mean and standard deviation by a factor of 0.5 and 2 and verifying that the parameter estimates were quantitatively similar |
| Auditory and visual location prior standard deviation ($\sigma_{ap},\sigma_{vp}$) | $\ln\sigma_{ap}\sim\ln\sigma_{a}+\zeta_{a}$  $\ln\sigma_{vp}\sim\ln\sigma_{a}+\zeta_{v}$  where  $\zeta_{a}\sim\mathcal{N(}{0,1}^{2})$  $\zeta_{v}\sim\mathcal{N}\left( {0,1}^{2} \right)$ | While combining the prior information about the cue locations with the likelihood, the weight on the prior/likelihood depends on the ratio of the likelihood and prior variances (refer $\alpha_{a}$ and $\alpha_{v}$ in Eq. S2). In such a case, to ensure that the same range of $\alpha$ are a-priori likely; independent of the specific value of $\sigma_{a}$, the prior over the auditory prior is varied around $\sigma_{a}$ in logarithmic space |
| Visual location sensory noise standard deviation (one parameter $\sigma_{vr}$for each reliability r) | $\ln\sigma_{v,r}\sim\ln\sigma_{a}+\zeta_{v,r}$  where  $\zeta_{v,r}\sim\mathcal{N}\left( {0,1}^{2} \right)$ | Same motivation as for the auditory and visual prior (see above) as the when combining the auditory and visual cues, the weights given to either cue depends on the ratio of their relative variances. (refer $\alpha_{a'v'}$ in Eq. S2). |
| p_common_ | $\text{p}\text{common}\sim\mathcal{U}\left( 0,1 \right)$ | Since this is the main parameter of interest, we use a flat prior to prevent any biases due to the prior |
| Choice bias (auditory discrimination$p_{\text{choice}}^{\text{aud}}$) | $p_{\text{choice}}^{\text{aud}}\sim Beta(1.25,1.25)$ | Weakly informative prior preferring unbiased responses |
| Choice bias (visual discrimination $p_{\text{choice}}^{\text{vis}}$, multisensory discrimination $p_{\text{choice}}^{\text{multi}}$, implicit causal inference $p_{\text{choice}}^{\text{implicit}}$) | $p_{\text{choice}}^{\text{aud}}\sim Sigmoid[\ln\frac{p_{\text{choice}}^{\text{aud}}}{1-p_{\text{choice}}^{\text{aud}}}+\zeta_{vis}$]  $p_{\text{choice}}^{\text{vis}}\sim Sigmoid[\ln\frac{p_{\text{choice}}^{\text{aud}}}{1-p_{\text{choice}}^{\text{aud}}}+\zeta_{multi}$]  $p_{\text{choice}}^{\text{multi}}\sim Sigmoid[\ln\frac{p_{\text{choice}}^{\text{aud}}}{1-p_{\text{choice}}^{\text{aud}}}+\zeta_{implicit}$]  where  $\zeta_{vis},\zeta_{multi},\zeta_{implicit}\sim\mathcal{N(}{0,0.5}^{2})$ | Hierarchical: Since all the mentioned tasks are discrimination tasks, this prior allows the model to hierarchically infer a choice that is consistent across experiments unless the data suggests otherwise |
| p_combined_(explicit causal inference) | $\text{p}\text{co}\text{mbined }\sim\mathcal{U}\left( 0,1 \right)$ | Since this is the main parameter of interest, we use a flat prior to avoid biases due to the prior |
