# Supplementary material for: Aberrant causal inference and presence of a compensatory mechanism in autism spectrum disorder
Source: eLife. 2022 May 17;11:e71866. doi: 10.7554/eLife.71866 (PMC9170250; doi:10.7554/eLife.71866)
Supplement: Supplementary file 4. [file elife-71866-supp4.docx]

**Supplementary File 4. Causal inference model inference and fitting details**

In order to infer the posterior over D_imp_ in the implicit task (Eq. 14), we can combine Eqs. 5,6 and 11 to get the likelihood over D_imp_ and C by integrating over all inferred cue location values as follows,

$p_{implicit}\left( X_{a},X_{v}|D_{\text{imp}},C \right)\propto\iint p\left( X_{a} | S_{a} \right)p\left( X_{v} | S_{v} \right)p_{implicit}\left( S_{a},S_{v}|D_{\text{imp}},C \right)dS_{a}dS_{v}$ **(Eq. S1)**

We can expand Eq. S1 using rules for the product of Gaussian probability density functions to get,

$$p_{implicit}\left( X_{a},X_{v}|D_{\text{imp}},C \right)\propto\left( C-1 \right)\Phi\left[ \frac{D_{\text{imp}}\left( X_{a}\alpha_{a}+\mu_{a}\left( 1-\alpha_{a} \right) \right)}{\sigma_{a}\sqrt{\alpha_{a}}} \right]$$

$+\left( 2-C \right)\mathcal{N}\left( X_{a'};X_{v'},\sigma_{a}^{2}\alpha_{a}+\sigma_{v}^{2}\alpha_{v} \right)\Phi\left[ \frac{D_{\text{imp}}\left( X_{a'}\alpha_{a^{'}v^{'}} +X_{v'}{(1-\alpha}_{a^{'}v^{'}}) \right)}{\sigma_{a}\sqrt{{\alpha_{a^{'}v^{'}}\alpha}_{a}}} \right]$ **(Eq. S2)**

where $\alpha_{a}=\frac{\sigma_{ap}^{2}}{\sigma_{a}^{2}+\sigma_{ap}^{2}}$, $\alpha_{v}=\frac{\sigma_{vp}^{2}}{\sigma_{v}^{2}+\sigma_{vp}^{2}}$, $X_{a'}=X_{a}\alpha_{a}+\mu_{a}\left( 1-\alpha_{a} \right)$, $X_{v'}=X_{v}\alpha_{v}+\mu_{v}\left( 1-\alpha_{v} \right)$ and $\alpha_{a'v'}=\frac{\sigma_{v}^{2}\alpha_{v}}{\sigma_{a}^{2}\alpha_{a}+\sigma_{v}^{2}\alpha_{v}}$

which we can evaluate using the previous equations as

$p_{implicit}\left( D_{\text{imp}}=1 | X_{a},X_{v},C=c \right)\propto p_{implicit}(X_{a},X_{v}|D_{\text{imp}}=1,C=c)p(C=c|D=1)p_{implicit}(D_{\text{imp}}=1)$ **Eq. (S3)**

and

$p(C=c|X_{a},X_{v})\propto\sum_{d\in\left\{ -1,1 \right\}} p_{implicit}\left( X_{a},X_{v} | D_{\text{imp}}=d,C=c \right)p(C=c|D_{\text{imp}}=d)p_{implicit}(D_{\text{imp}}=d)$ **(Eq. S4)**

The subject uses the inferred posterior probability over D_imp_ to make their choice by comparing which response has a higher probability of being correct (Eq. 14). Since D_imp_ is a binary variable, this is equivalent to checking if $p\left( D_{\text{imp}}=1 | X_{a},X_{v} \right)>0.5$. Since we do not have access to the subject’s sensory observations, we marginalize over all possible sensory observations using Eqs. 3 and 4. We also model lapses in subject judgements using a lapse rate (denoted by $p_{\text{lapse rate}}^{\text{imp}}$) and lapse bias (denoted by $p_{\text{lapse bias}}^{\text{imp}}$). Lapse rate characterizes the probability that the subject makes a choice independent of the stimulus. The lapse response is modeled as a random sample from a Bernoulli distribution with parameter $p_{\text{lapse rate}}^{\text{imp}}.$ Mathematically, the probability of the subject making a choice $R_{\text{implicit}}$=1 (equivalently for $R_{\text{explicit}}$=1) for a given set of experimenter defined $\epsilon_{a}$ and $\epsilon_{v}$ is given by,

$p\left( R_{\text{implicit}}=1 | \epsilon_{a},\epsilon_{v} \right)=p_{\text{lapse rate}}^{\text{imp}}p_{\text{lapse bias}}^{\text{imp}}+\left( 1-p_{\text{lapse rate}}^{\text{imp}} \right)\iint H[p\left( D_{\text{imp}}=1 | X_{a},X_{v} \right)-0.5]p\left( X_{a} | \epsilon_{a} \right)p\left( X_{v} | \epsilon_{v} \right)dX_{a}dX_{v}$

**(Eq. S5)**

We perform the integral in Eq. S5 for the implicit task by numerically evaluating the decision boundary using a multi-dimensional bisection method (Bachrathy et al. 2012) and then using this to evaluate the integral analytically (the complete analytic solution is unavailable). This provides a better estimate than traditional sampling or numerical methods since the decision function is not smooth. To verify the estimates, we also obtained estimates by numerical integration using gaussian quadrature (Golub et al. 1969). We use analytic solutions for the explicit task.

For the aggregate subject model predictions, we found the Maximum a Posteriori (MAP) estimate for the model parameters under weakly informative priors for the sensory parameters and choice parameters (details in the *Supplementary Material S6*). The fitting was done using a quasi-newton Broyden-Fletcher-Goldfarb-Shanno (BFGS) unconstrained optimization procedure (fminunc in MATLAB) using 40 restarts to find the global minimum. We obtained full the posterior over the parameters for the aggregate control subjects using generalized elliptical slice sampling (Nishihara et al. 2014) which allowed us to quantify the uncertainty over the parameter estimates. Since our model prediction for ASD subjects requires setting the sensory parameters to the same value as the control and explaining the differences in terms of the choice and inference parameters, we obtained posterior estimates for the choice and inference parameters for $n_{approx}$ independent samples from the control parameter fits (184 in our case) and then computed the effective mean and standard deviation across each posterior to get the uncertainty around the fitted ASD parameters. The posterior over the ASD parameters obtained using the method described above is mathematically illustrated below

$$p\left( \theta_{ASD}^{'} | \mathcal{D}_{control},\mathcal{D}_{ASD} \right)=\ldots\iiint p\left( \theta_{ASD}^{'} | \mathcal{D}_{control},\mathcal{D}_{ASD},\theta_{control}^{''} \right)p\left( \theta_{control}^{''} | \mathcal{D}_{control} \right)d\theta_{control}^{''}$$

$p\left( \theta_{ASD}^{'} | \mathcal{D}_{control},\mathcal{D}_{ASD} \right)\approx\frac{1}{n_{approx}}\sum_{\theta_{control}^{''\left( i \right)}\sim p\left( \theta_{control}^{''} | \mathcal{D}_{control} \right)} p\left( \theta_{ASD}^{'} | \mathcal{D}_{control},\mathcal{D}_{ASD},\theta_{control}^{''\left( i \right)} \right)$**(Eq. S6)**

where $\theta_{ASD}^{'}$ are the choice and inference parameters for the aggregate ASD subject, $\theta_{control}^{''}$ are the sensory parameters for the aggregate control subject, $\mathcal{D}_{ASD},\mathcal{D}_{control}$ are ASD and control data.

In order get a goodness of fit estimate of the model, we computed the explainable variance explained (EVE, Haefner et al. 2008). This estimate is an extension of the tradition variance explained/ coefficient of determination but accounts for the uncertainty in the data generation process which is the case for us with limited number of trials per condition. In addition, it corrects for the number of parameters in the model to allow for overfitting.

We also obtained full posteriors over model parameters for the individual subjects by jointly fitting the model to all experiments with weakly informative priors. We modeled each observer population with a hierarchical model where an individual observer’s parameters are independent draws from the population parameter, parameterized as a Gaussian, i.e. $\theta_{subject}\sim\mathcal{N}(\theta_{population},\sigma_{population}^{2})$ where $\theta$ are the parameters of the causal inference model. We also approximated the posterior over the subject parameters as Gaussians which allowed us to analytically combine the individual posteriors to a combined Gaussian population posterior for further hypothesis testing and obtaining confidence intervals (CI). For the individual subject estimates, we plot 68%CI. We obtained the p-value for the difference between ASD and control subjects parameters using two methods: (a) Normal approximated analytical p-value which assumes that the sample variance is equal to the population variance, (b) Welch t-test which relaxes the above assumption. Both methods gave comparable p-values and we conservatively considered the higher p-value for significance testing.
