# Supplementary material for: Aberrant causal inference and presence of a compensatory mechanism in autism spectrum disorder
Source: eLife. 2022 May 17;11:e71866. doi: 10.7554/eLife.71866 (PMC9170250; doi:10.7554/eLife.71866)
Supplement: Supplementary file 5. [file elife-71866-supp5.docx]

**Supplementary File 5. Causal inference modeling for simultaneity judgements**

We modeled the explicit simultaneity judgment task using a causal inference model where the subject infers the temporal disparity between the auditory and visual cues and reports whether the two cues occurred at the same time or not. Instead of characterizing temporal judgements in units of veridical time, which restricts the shape of sensory uncertainty and priors (to accurately model the non-reversible property of veridical time), we can measure the temporal judgements with respect to a reference cue which can be positive or negative. We choose the reference cue (without loss of generality) as the time of the auditory cue and therefore the full causal inference model reduced to a model over the perceived disparity, where inferring the two cues as coming from the same cause is the same as inferring if the disparity is zero or not. The derivation of the model is similar to the one presented in the main text for spatial judgements. The observed disparity is modeled as the true disparity corrupted by Gaussian sensory noise:

$p\left( X_{t} | \epsilon_{t} \right)\mathcal{= N(}X_{t};\epsilon_{t},\sigma_{t}^{2})$ **(Eq. S7)**

As before, we assume that subjects have a good estimate of their sensory uncertainty (from lifelong learning) and therefore the likelihood of the inferred disparity percept is the same as Eq. S7

$l\left( S_{t} \right)\equiv p\left( X_{t} | S_{t} \right)\mathcal{= N(}X_{t};S_{t},\sigma_{t}^{2})$ **(Eq. S8)**

The prior over temporal disparities is parametrized as a Gaussian modulation by whether the subject infers the disparity as zero or not.

$p(S_{t}|C)= \mathcal{N}\left( S_{t};\mu_{t},\sigma_{p}^{2} \right)\left[ \left( C-1 \right)+\left( 2-C \right)\delta\left( S_{t} \right) \right]$ **(Eq. S9)**

The prior probability of the subject’s belief over D_temp_ in the temporal task is parameterized a Bernoulli distribution with mean $p_{\text{choice}}^{\text{temporal}}$ as given in Eq. S10

$p_{\text{temporal}}(D_{\text{temp}}=1)= Ber(D_{\text{temp}};p_{\text{choice}}^{\text{temporal}})$ **(Eq. S10)**

The influence of the inferred trial category (D_temp_) on the common cause (C) is characterized by a modulation due to task learning (parameterized by $\alpha_{\text{task}}$) as defined in Eq. S11 and S12

$p(C=1|D_{\text{temp}}=1)= Ber[C;p_{\text{common}}^{\text{temporal}}+\alpha_{\text{task}}^{\text{temporal}}\left( 1-p_{\text{common}}^{\text{temporal}} \right)]$ **(Eq. S11)**

$p(C=1|D_{\text{temp}}=2)= Ber[C;p_{\text{common}}^{\text{temporal}}-\alpha_{\text{task}}^{\text{temporal}}\left( p_{\text{common}}^{\text{temporal}} \right)]$ **(Eq. S12)**

The subject uses the inferred posterior probability over D_temp_ to make their choice by comparing which value of D_temp_ has a higher probability (i.e., ideal observer). The posterior probability can be obtained using Bayes rule and marginalizing across $S_{t}$

$p\left( D_{\text{temp}}=1 | X_{t} \right)\propto\sum_{C} \int p\left( X_{t} | S_{t} \right)p\left( S_{t} | C \right)p(C|D_{\text{temp}})p_{temporal}\left( D_{\text{temp}}=1 \right)dS_{t}$ **(Eq. S13)**

The subject therefore makes a choice $R_{\text{temporal}}$=1 if $p\left( D_{\text{temp}}=1 | X_{t} \right)>p\left( D_{\text{temp}}=2 | X_{t} \right)$ which can be expanded using Bayes rule as

$p(X_{t}|D_{\text{temp}}=1)p_{\text{temporal}}(D_{\text{temp}}=1)>p(X_{t}|D_{\text{temp}}=2)p_{\text{temporal}}(D_{\text{temp}}=2)$ **(Eq. S14)**

Using Eqs. S8-S13, we can expand S14 (similar to the derivation of the explicit task in the main text) to get the decision rule as

$p(X_{t}|C=1)p_{\text{combined}}^{\text{temporal}}>p(X_{t}|C=2)p_{\text{combined}}^{\text{temporal}}$ **(Eq. S15)**

where

$p_{\text{combined}}^{\text{temporal}}=\max(0,\min(1,\frac{p_{\text{common}}^{\text{temporal}}{(2p}_{\text{choice}}^{\text{temporal}}-1)+\alpha_{\text{task}}[p_{\text{common}}^{\text{temporal}}(1-p_{\text{choice}}^{\text{temporal}})+p_{\text{choice}}^{\text{temporal}}(1-p_{\text{common}}^{\text{temporal}})]}{\text{(2}p_{\text{common}}^{\text{temporal}}\text{ }-1)(2p_{\text{choice}}^{\text{temporal}}-1)+2\alpha_{\text{task}}[p_{\text{common}}^{\text{temporal}}(1-p_{\text{choice}}^{\text{temporal}})+p_{\text{choice}}^{\text{temporal}}(1-p_{\text{common}}^{\text{temporal}})]}))$ **(Eq. S16)**

We can see from Eq. S16 that, as in the case of the spatial explicit task, $p_{\text{combined}}^{\text{temporal}}$ which a function of $\alpha_{\text{task}}^{\text{temporal}}$,$p_{\text{common}}^{\text{temporal}}$ and $p_{\text{choice}}^{\text{temporal}}$ cannot be individually constrained from the subject responses.

Since we do not have access to the subject’s sensory observations, we marginalize over all possible sensory observations using Eq. S7. We also model lapses in subject judgements using a lapse rate (denoted by $p_{\text{lapse rate}}^{\text{temp}}$) and lapse bias (denoted by $p_{\text{lapse bias}}^{\text{temp}}$). Lapse rate characterizes the probability that the subject makes a stimulus independent choice. The lapse response is modeled as a random sample from a Bernoulli distribution with a parameter $p_{\text{lapse rate}}^{\text{temp}}.$ Mathematically, the probability of the subject making a choice $R_{\text{temporal}}$=1 for a given set of experimenter defined $\epsilon_{a}$ and $\epsilon_{v}$ is given by,

$p\left( R_{\text{temporal}}=1 | \epsilon_{t} \right)=p_{\text{lapse rate}}^{\text{temp}}p_{\text{lapse bias}}^{\text{temp}}+\left( 1-p_{\text{lapse rate}}^{\text{temp}} \right)\iint H[p\left( D_{\text{temp}}=1 | X_{t} \right)-0.5]p\left( X_{t} | \epsilon_{t} \right)dX_{a}dX_{v}$ **(Eq. S17)**
