# Supplementary material for: Aberrant causal inference and presence of a compensatory mechanism in autism spectrum disorder
Source: eLife. 2022 May 17;11:e71866. doi: 10.7554/eLife.71866 (PMC9170250; doi:10.7554/eLife.71866)
Supplement: Transparent reporting form [file elife-71866-transrepform1.pdf]

eLIFE
1st Floor
24 Hills Road
Cambridge CB2 1JP, UK
P 01223 855340
W elifesciences.org
T @elife

eLife's transparent reporting form

We encourage authors to provide detailed information *within their submission* to facilitate the interpretation and replication of experiments. Authors can upload supporting documentation to indicate the use of appropriate reporting guidelines for health-related research (see EQUATOR Network), life science research (see the BioSharing Information Resource), or the ARRIVE guidelines for reporting work involving animal research. Where applicable, authors should refer to any relevant reporting standards documents in this form.

If you have any questions, please consult our Journal Policies and/or contact us: editorial@elifesciences.org.

Sample-size estimation

- You should state whether an appropriate sample size was computed when the study was being designed
- You should state the statistical method of sample size computation and any required assumptions
- If no explicit power analysis was used, you should describe how you decided what sample (replicate) size (number) to use

Please outline where this information can be found within the submission (e.g., sections or figure legends), or explain why this information doesn't apply to your submission:

This information can be found in the Materials and Methods section, under Participants. The relevant text is:

For each of the 4 psychophysics experiments we aimed at scheduling approximately 25-30 participants per group, in accord with samples sizes from previous similar reports (Dokka et al., 2019; Noel et al., 2018b). Data were not examined until after data collection was complete

Replicates

- You should report how often each experiment was performed
- You should include a definition of biological versus technical replication
- The data obtained should be provided and sufficient information should be provided to indicate the number of independent biological and/or technical replicates
- If you encountered any outliers, you should describe how these were handled
- Criteria for exclusion/inclusion of data should be clearly stated
- High-throughput sequence data should be uploaded before submission, with a private link for reviewers provided (these are available from both GEO and ArrayExpress)

Please outline where this information can be found within the submission (e.g., sections or figure legends), or explain why this information doesn't apply to your submission:

eLife Sciences Publications, Ltd is a limited liability non-profit non-stock corporation incorporated in the State of Delaware, USA, with company number 5030732, and is registered in the UK with company number FC030576 and branch number BR015634 at the address 1st Floor, 24 Hills Road, Cambridge CB2 1JP | August 2014

This information can be found throughout the main text. Two examples where outlier/data removal are clearly stated are:

Auditory discrimination seemed to highlight potentially two subgroups within the ASD cohort (blue vs. green). Auditory threshold estimation was not possible for 6 of the 31 subjects within the ASD group (**Fig. 1D,** green, R^2 value < 0.50), due to a lack of modulation in their reports as a function of cue location (excluding these 6 subjects, average R^2 ASD = 0.95; average R^2 ASD = 0.96).

And:

As expected, most subjects reported a common source more frequently at smaller rather than larger Δ (**Fig. 3A-C**, F(8, 259) = 94.86, p < 0.001). Interestingly, while this pattern was true for all individual control subjects, 8 of the individuals with ASD (i.e., ~1/3) did not modulate their explicit common cause reports as a function of spatial disparity, despite good auditory and visual localization (see **Fig. S1**). These subjects were not included in subsequent analyses. As visual reliability worsened (**Fig. 3**, from **A-C)**, both groups reported common cause less frequently (F(2, 74) = 10.68, p < 0.001).

Statistical reporting

- Statistical analysis methods should be described and justified
- Raw data should be presented in figures whenever informative to do so (typically when N per group is less than 10)
- For each experiment, you should identify the statistical tests used, exact values of N, definitions of center, methods of multiple test correction, and dispersion and precision measures (e.g., mean, median, SD, SEM, confidence intervals; and, for the major substantive results, a measure of effect size (e.g., Pearson's r, Cohen's d)
- Report exact p-values wherever possible alongside the summary statistics and 95% confidence intervals. These should be reported for all key questions and not only when the p-value is less than 0.05.

Please outline where this information can be found within the submission (e.g., sections or figure legends), or explain why this information doesn't apply to your submission:

This information can be found throughout the results and methods sections. We describe explicitly each statistical test, and report exact p-values.

(For large datasets, or papers with a very large number of statistical tests, you may upload a single table file with tests, Ns, etc., with reference to sections in the manuscript.)

Group allocation

- Indicate how samples were allocated into experimental groups (in the case of clinical studies, please specify allocation to treatment method); if randomization was used, please also state if restricted randomization was applied
- Indicate if masking was used during group allocation, data collection and/or data analysis

Please outline where this information can be found within the submission (e.g., sections or figure legends), or explain why this information doesn't apply to your submission:

eLife Sciences Publications, Ltd is a limited liability non-profit non-stock corporation incorporated in the State of Delaware, USA, with company number 5030732, and is registered in the UK with company number FC030576 and branch number BR015634 at the address 1st Floor, 24 Hills Road, Cambridge CB2 1JP | August 2014

This is indicated in the participants section, as follows:

A total of 91 adolescents (16.25 ± 0.4 years; 20 females) took part (completely or partially) in a series of up to 4 behavioral experiments. Forty-four (16.0 ± 0.5 years; 7 females) of these participants were diagnosed as within the Autism Spectrum Disorder (ASD). Inclusion in the ASD group required that subjects have (1) a confirmed diagnosis of ASD by a research-reliable clinical practitioner and (2) no history of seizure or other neurological disorders. Intelligence Quotient (IQ) was available for a subset of the ASD participants, whose average score was 103 ± 9 (S.E.M.), this being no different from the general population (which by definition has a mean of 100). Participants with ASD were recruited through several sources, including the Simons Simplex Collection families, flyers posted at Texas Children's Hospital, the Houston Autism Center, diverse learning centers for children diagnosed with ASD, and the clinical databases maintained by the Simons Foundation for Autism Research. The other 47 participants (16.5 ± 0.4 years; 13 females) were neurotypical controls. Individuals in the control group had no diagnosis of ASD or any other developmental disorder or related medical diagnosis. All participants were screened at enrollment with the Social Communication Questionnaire (SCQ; Rutter et al. 2003) and/or the Autism Questionnaire (AQ; Baron-Cohen et al. 2001) to afford (1) a measure of current ASD symptomatology and (2) rule out concerns for ASD in control subjects.

Additional data files ("source data")

- We encourage you to upload relevant additional data files, such as numerical data that are represented as a graph in a figure, or as a summary table
- Where provided, these should be in the most useful format, and they can be uploaded as "Source data" files linked to a main figure or table
- Include model definition files including the full list of parameters used
- Include code used for data analysis (e.g., R, MatLab)
- Avoid stating that data files are "available upon request"

Please indicate the figures or tables for which source data files have been provided:

There is a data and code availability section in the methods indicating that:
Data and code are available at https://osf.io/6xbzt.
